# 2-Phenoxy­pyrimidine

**DOI:** 10.1107/S1600536808041196

**Published:** 2008-12-13

**Authors:** Nasir Shah Bakhtiar, Zanariah Abdullah, Seik Weng Ng

**Affiliations:** a Department of Chemistry, University of Malaya, 50603 Kuala Lumpur, Malaysia

## Abstract

There are two molecules in the asymmetric unit of, C_10_H_8_N_2_O, with dihedral angles between the aromatic ring planes of 75.9 (1) and 79.3 (1)°.

## Related literature

For other phen­oxy-substituted *N*-heterocycles, see: Abdullah & Ng (2008 ▶); Hassan *et al.* (2008 ▶); Idris *et al.* (2009 ▶).
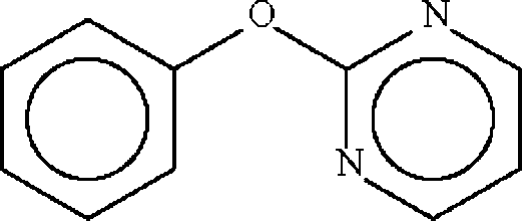


## Experimental

### 

#### Crystal data

C_10_H_8_N_2_O
*M* *_r_* = 172.18
Monoclinic, 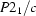

*a* = 10.859 (1) Å
*b* = 20.181 (2) Å
*c* = 8.1339 (8) Å
β = 106.637 (2)°
*V* = 1707.8 (3) Å^3^
*Z* = 8
Mo *K*α radiation
μ = 0.09 mm^−1^
*T* = 100 (2) K
0.25 × 0.20 × 0.15 mm

#### Data collection

Bruker SMART APEX diffractometer
Absorption correction: none
9752 measured reflections
3901 independent reflections
3026 reflections with *I* > 2σ(*I*)
*R*_int_ = 0.024

#### Refinement

*R*[*F* ^2^ > 2σ(*F* ^2^)] = 0.037
*wR*(*F* ^2^) = 0.102
*S* = 1.03
3901 reflections
235 parameters
H-atom parameters constrained
Δρ_max_ = 0.18 e Å^−3^
Δρ_min_ = −0.22 e Å^−3^

### 

Data collection: *APEX2* (Bruker, 2007 ▶); cell refinement: *SAINT* (Bruker, 2007 ▶); data reduction: *SAINT*; program(s) used to solve structure: *SHELXS97* (Sheldrick, 2008 ▶); program(s) used to refine structure: *SHELXL97* (Sheldrick, 2008 ▶); molecular graphics: *X-SEED* (Barbour, 2001 ▶); software used to prepare material for publication: *publCIF* (Westrip, 2009 ▶).

## Supplementary Material

Crystal structure: contains datablocks global, I. DOI: 10.1107/S1600536808041196/tk2342sup1.cif

Structure factors: contains datablocks I. DOI: 10.1107/S1600536808041196/tk2342Isup2.hkl

Additional supplementary materials:  crystallographic information; 3D view; checkCIF report

## Figures and Tables

**Fig. 1.**
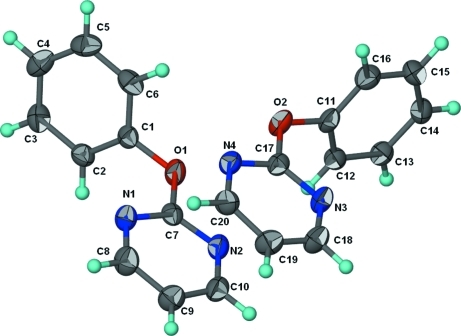
Thermal ellipsoid plot (Barbour, 2001) of the two independent molecules of C10H8N2O at the 70% probability level. Hydrogen atoms are drawn as spheres of arbitrary radius.

## supplementary crystallographic information

### Experimental

Phenol (1.88 g, 20 mmol) was mixed with sodium hydroxide (0.08 g, 20 mmol) in several drops of water. The water was then evaporated. The paste was heated with 2-chloropyrimidine (2.30 g, 20 mmol) at 423–433 K for 6 h. The product was dissolved in water and the solution extracted with ether. The ether phase was dried over sodium sulfate; the evaporation of the solvent gave well shaped colorless crystals along with some unidentified brown material.

### Refinement

Carbon-bound H-atoms were placed in calculated positions (C—H 0.95 Å) and were included in the refinement in the riding model approximation, with *U*(H) set to 1.2*U*_eq_(C).

### Crystal data

**Table:**

C_10_H_8_N_2_O	*F*(000) = 720
*M**_r_* = 172.18	*D*_x_ = 1.339 Mg m^−^^3^
Monoclinic, *P*2_1_/*c*	Mo *K*α radiation, λ = 0.71073 Å
Hall symbol: -P 2ybc	Cell parameters from 2782 reflections
*a* = 10.859 (1) Å	θ = 2.2–28.2°
*b* = 20.181 (2) Å	µ = 0.09 mm^−^^1^
*c* = 8.1339 (8) Å	*T* = 100 K
β = 106.637 (2)°	Block, colorless
*V* = 1707.8 (3) Å^3^	0.25 × 0.20 × 0.15 mm
*Z* = 8	

### Data collection

**Table:**

Bruker SMART APEX diffractometer	3026 reflections with *I* > 2σ(*I*)
Radiation source: fine-focus sealed tube	*R*_int_ = 0.024
graphite	θ_max_ = 27.5°, θ_min_ = 2.0°
ω scans	*h* = −11→14
9752 measured reflections	*k* = −26→26
3901 independent reflections	*l* = −10→10

### Refinement

**Table:**

Refinement on *F*^2^	Primary atom site location: structure-invariant direct methods
Least-squares matrix: full	Secondary atom site location: difference Fourier map
*R*[*F*^2^ > 2σ(*F*^2^)] = 0.037	Hydrogen site location: inferred from neighbouring sites
*wR*(*F*^2^) = 0.102	H-atom parameters constrained
*S* = 1.03	*w* = 1/[σ^2^(*F*_o_^2^) + (0.0497*P*)^2^ + 0.2571*P*] where *P* = (*F*_o_^2^ + 2*F*_c_^2^)/3
3901 reflections	(Δ/σ)_max_ = 0.001
235 parameters	Δρ_max_ = 0.18 e Å^−^^3^
0 restraints	Δρ_min_ = −0.22 e Å^−^^3^

### Fractional atomic coordinates and isotropic or equivalent isotropic displacement parameters (Å^2^)

**Table:**

	*x*	*y*	*z*	*U*_iso_*/*U*_eq_	
O1	0.69295 (8)	0.58216 (5)	0.51546 (11)	0.0274 (2)	
O2	0.97744 (9)	0.67233 (5)	0.77250 (11)	0.0281 (2)	
N1	0.59037 (10)	0.60351 (5)	0.72195 (13)	0.0245 (2)	
N2	0.77301 (10)	0.53229 (5)	0.76936 (14)	0.0253 (2)	
N3	1.05326 (11)	0.62385 (5)	1.03911 (13)	0.0264 (3)	
N4	0.87443 (10)	0.69812 (5)	0.96601 (13)	0.0247 (2)	
C1	0.59965 (12)	0.62116 (6)	0.40077 (16)	0.0213 (3)	
C2	0.48239 (12)	0.59386 (6)	0.31580 (17)	0.0256 (3)	
H2	0.4617	0.5500	0.3408	0.031*	
C3	0.39513 (13)	0.63124 (7)	0.19343 (18)	0.0297 (3)	
H3	0.3136	0.6133	0.1346	0.036*	
C4	0.42700 (14)	0.69480 (7)	0.15706 (17)	0.0311 (3)	
H4	0.3671	0.7204	0.0729	0.037*	
C5	0.54526 (14)	0.72141 (6)	0.24218 (18)	0.0314 (3)	
H15A	0.5666	0.7650	0.2161	0.038*	
C6	0.63298 (13)	0.68443 (6)	0.36592 (17)	0.0263 (3)	
H6B	0.7144	0.7024	0.4254	0.032*	
C7	0.68331 (12)	0.57302 (6)	0.67684 (16)	0.0208 (3)	
C8	0.58755 (14)	0.59106 (7)	0.88258 (17)	0.0303 (3)	
H8	0.5227	0.6115	0.9225	0.036*	
C9	0.67458 (14)	0.54993 (7)	0.99239 (17)	0.0295 (3)	
H9	0.6711	0.5416	1.1059	0.035*	
C10	0.76711 (13)	0.52151 (6)	0.92888 (17)	0.0267 (3)	
H10	0.8291	0.4931	1.0015	0.032*	
C11	1.07433 (12)	0.63814 (6)	0.72453 (15)	0.0235 (3)	
C12	1.05906 (12)	0.57148 (6)	0.68472 (15)	0.0235 (3)	
H12	0.9859	0.5480	0.6954	0.028*	
C13	1.15275 (12)	0.53963 (6)	0.62894 (15)	0.0239 (3)	
H13	1.1439	0.4939	0.6007	0.029*	
C14	1.25931 (13)	0.57413 (7)	0.61407 (16)	0.0258 (3)	
H14	1.3239	0.5518	0.5773	0.031*	
C15	1.27177 (13)	0.64082 (7)	0.65256 (18)	0.0298 (3)	
H15	1.3445	0.6644	0.6409	0.036*	
C16	1.17853 (13)	0.67363 (6)	0.70825 (17)	0.0284 (3)	
H16	1.1865	0.7196	0.7345	0.034*	
C17	0.96950 (12)	0.66360 (6)	0.93451 (15)	0.0214 (3)	
C18	1.03893 (14)	0.61819 (7)	1.19692 (17)	0.0302 (3)	
H18	1.0957	0.5900	1.2774	0.036*	
C19	0.94557 (14)	0.65152 (7)	1.24657 (16)	0.0293 (3)	
H19	0.9368	0.6472	1.3590	0.035*	
C20	0.86503 (13)	0.69161 (6)	1.12499 (17)	0.0271 (3)	
H20	0.7999	0.7157	1.1560	0.033*	

### Atomic displacement parameters (Å^2^)

**Table:**

	*U*^11^	*U*^22^	*U*^33^	*U*^12^	*U*^13^	*U*^23^
O1	0.0224 (5)	0.0355 (5)	0.0264 (5)	0.0089 (4)	0.0102 (4)	0.0033 (4)
O2	0.0304 (5)	0.0329 (5)	0.0221 (5)	0.0140 (4)	0.0092 (4)	0.0064 (4)
N1	0.0230 (6)	0.0253 (5)	0.0255 (6)	0.0043 (4)	0.0075 (5)	−0.0019 (4)
N2	0.0205 (6)	0.0237 (5)	0.0302 (6)	0.0025 (4)	0.0050 (5)	−0.0001 (4)
N3	0.0246 (6)	0.0298 (6)	0.0219 (5)	0.0066 (5)	0.0018 (4)	0.0021 (4)
N4	0.0242 (6)	0.0241 (5)	0.0261 (6)	0.0051 (4)	0.0079 (5)	0.0030 (4)
C1	0.0202 (6)	0.0238 (6)	0.0221 (6)	0.0030 (5)	0.0097 (5)	−0.0010 (5)
C2	0.0237 (7)	0.0206 (6)	0.0340 (7)	−0.0018 (5)	0.0107 (6)	0.0004 (5)
C3	0.0232 (7)	0.0314 (7)	0.0326 (7)	0.0008 (5)	0.0052 (6)	−0.0040 (6)
C4	0.0389 (8)	0.0280 (7)	0.0260 (7)	0.0102 (6)	0.0084 (6)	0.0020 (5)
C5	0.0441 (9)	0.0198 (6)	0.0346 (7)	−0.0002 (6)	0.0181 (7)	0.0007 (5)
C6	0.0262 (7)	0.0263 (6)	0.0291 (7)	−0.0069 (5)	0.0121 (6)	−0.0077 (5)
C7	0.0192 (6)	0.0181 (5)	0.0248 (6)	−0.0026 (5)	0.0059 (5)	−0.0031 (5)
C8	0.0297 (7)	0.0349 (7)	0.0285 (7)	0.0062 (6)	0.0118 (6)	−0.0031 (6)
C9	0.0321 (8)	0.0323 (7)	0.0231 (6)	0.0014 (6)	0.0059 (6)	0.0008 (5)
C10	0.0239 (7)	0.0236 (6)	0.0288 (7)	0.0007 (5)	0.0015 (5)	0.0012 (5)
C11	0.0232 (7)	0.0282 (6)	0.0175 (6)	0.0075 (5)	0.0034 (5)	0.0034 (5)
C12	0.0213 (6)	0.0276 (6)	0.0209 (6)	0.0000 (5)	0.0052 (5)	0.0034 (5)
C13	0.0246 (7)	0.0248 (6)	0.0205 (6)	0.0010 (5)	0.0037 (5)	−0.0004 (5)
C14	0.0218 (7)	0.0313 (7)	0.0240 (6)	0.0039 (5)	0.0064 (5)	0.0011 (5)
C15	0.0226 (7)	0.0320 (7)	0.0345 (7)	−0.0030 (6)	0.0077 (6)	0.0028 (6)
C16	0.0298 (7)	0.0234 (6)	0.0292 (7)	0.0006 (5)	0.0039 (6)	0.0005 (5)
C17	0.0218 (6)	0.0202 (6)	0.0204 (6)	0.0007 (5)	0.0031 (5)	0.0000 (5)
C18	0.0308 (8)	0.0343 (7)	0.0210 (6)	0.0057 (6)	0.0001 (6)	0.0038 (5)
C19	0.0357 (8)	0.0316 (7)	0.0200 (6)	0.0016 (6)	0.0069 (6)	0.0015 (5)
C20	0.0275 (7)	0.0268 (6)	0.0291 (7)	0.0024 (5)	0.0113 (6)	0.0007 (5)

### Geometric parameters (Å, °)

**Table:**

O1—C7	1.3590 (15)	C6—H6B	0.9500
O1—C1	1.4053 (15)	C8—C9	1.3771 (19)
O2—C17	1.3565 (14)	C8—H8	0.9500
O2—C11	1.4037 (15)	C9—C10	1.3793 (19)
N1—C7	1.3205 (16)	C9—H9	0.9500
N1—C8	1.3394 (17)	C10—H10	0.9500
N2—C7	1.3307 (16)	C11—C16	1.3772 (19)
N2—C10	1.3352 (17)	C11—C12	1.3824 (18)
N3—C17	1.3235 (16)	C12—C13	1.3850 (17)
N3—C18	1.3414 (17)	C12—H12	0.9500
N4—C17	1.3297 (16)	C13—C14	1.3851 (18)
N4—C20	1.3324 (16)	C13—H13	0.9500
C1—C2	1.3780 (18)	C14—C15	1.3797 (18)
C1—C6	1.3785 (17)	C14—H14	0.9500
C2—C3	1.3848 (19)	C15—C16	1.3897 (19)
C2—H2	0.9500	C15—H15	0.9500
C3—C4	1.3824 (19)	C16—H16	0.9500
C3—H3	0.9500	C18—C19	1.3703 (19)
C4—C5	1.381 (2)	C18—H18	0.9500
C4—H4	0.9500	C19—C20	1.3792 (18)
C5—C6	1.389 (2)	C19—H19	0.9500
C5—H15A	0.9500	C20—H20	0.9500
			
C7—O1—C1	118.48 (9)	N2—C10—C9	122.61 (12)
C17—O2—C11	117.86 (9)	N2—C10—H10	118.7
C7—N1—C8	114.61 (11)	C9—C10—H10	118.7
C7—N2—C10	114.78 (11)	C16—C11—C12	122.03 (12)
C17—N3—C18	114.85 (11)	C16—C11—O2	118.22 (11)
C17—N4—C20	114.51 (11)	C12—C11—O2	119.62 (12)
C2—C1—C6	121.77 (12)	C11—C12—C13	118.57 (12)
C2—C1—O1	119.74 (11)	C11—C12—H12	120.7
C6—C1—O1	118.26 (11)	C13—C12—H12	120.7
C1—C2—C3	119.11 (12)	C12—C13—C14	120.38 (12)
C1—C2—H2	120.4	C12—C13—H13	119.8
C3—C2—H2	120.4	C14—C13—H13	119.8
C4—C3—C2	119.85 (13)	C15—C14—C13	120.04 (12)
C4—C3—H3	120.1	C15—C14—H14	120.0
C2—C3—H3	120.1	C13—C14—H14	120.0
C5—C4—C3	120.48 (13)	C14—C15—C16	120.37 (12)
C5—C4—H4	119.8	C14—C15—H15	119.8
C3—C4—H4	119.8	C16—C15—H15	119.8
C4—C5—C6	120.05 (12)	C11—C16—C15	118.61 (12)
C4—C5—H15A	120.0	C11—C16—H16	120.7
C6—C5—H15A	120.0	C15—C16—H16	120.7
C1—C6—C5	118.74 (12)	N3—C17—N4	128.48 (11)
C1—C6—H6B	120.6	N3—C17—O2	118.70 (11)
C5—C6—H6B	120.6	N4—C17—O2	112.82 (10)
N1—C7—N2	128.65 (11)	N3—C18—C19	122.58 (12)
N1—C7—O1	118.78 (11)	N3—C18—H18	118.7
N2—C7—O1	112.56 (10)	C19—C18—H18	118.7
N1—C8—C9	122.86 (12)	C18—C19—C20	116.58 (12)
N1—C8—H8	118.6	C18—C19—H19	121.7
C9—C8—H8	118.6	C20—C19—H19	121.7
C8—C9—C10	116.48 (12)	N4—C20—C19	123.00 (12)
C8—C9—H9	121.8	N4—C20—H20	118.5
C10—C9—H9	121.8	C19—C20—H20	118.5
			
C7—O1—C1—C2	−80.80 (14)	C17—O2—C11—C16	−106.60 (13)
C7—O1—C1—C6	104.66 (13)	C17—O2—C11—C12	77.48 (15)
C6—C1—C2—C3	−0.72 (19)	C16—C11—C12—C13	0.84 (19)
O1—C1—C2—C3	−175.06 (11)	O2—C11—C12—C13	176.60 (10)
C1—C2—C3—C4	0.64 (19)	C11—C12—C13—C14	0.14 (18)
C2—C3—C4—C5	−0.1 (2)	C12—C13—C14—C15	−0.92 (19)
C3—C4—C5—C6	−0.3 (2)	C13—C14—C15—C16	0.7 (2)
C2—C1—C6—C5	0.28 (18)	C12—C11—C16—C15	−1.03 (19)
O1—C1—C6—C5	174.70 (11)	O2—C11—C16—C15	−176.84 (11)
C4—C5—C6—C1	0.24 (19)	C14—C15—C16—C11	0.23 (19)
C8—N1—C7—N2	−0.11 (19)	C18—N3—C17—N4	0.05 (19)
C8—N1—C7—O1	179.36 (11)	C18—N3—C17—O2	179.77 (11)
C10—N2—C7—N1	−0.22 (19)	C20—N4—C17—N3	0.79 (19)
C10—N2—C7—O1	−179.71 (10)	C20—N4—C17—O2	−178.94 (11)
C1—O1—C7—N1	−3.06 (16)	C11—O2—C17—N3	0.36 (17)
C1—O1—C7—N2	176.49 (10)	C11—O2—C17—N4	−179.88 (10)
C7—N1—C8—C9	0.14 (19)	C17—N3—C18—C19	−0.63 (19)
N1—C8—C9—C10	0.1 (2)	N3—C18—C19—C20	0.3 (2)
C7—N2—C10—C9	0.52 (18)	C17—N4—C20—C19	−1.10 (19)
C8—C9—C10—N2	−0.5 (2)	C18—C19—C20—N4	0.6 (2)
